# Human-Animal Relationships in Supported Housing: Animal Atmospheres for Mental Health Recovery

**DOI:** 10.3389/fpsyg.2021.712133

**Published:** 2021-08-18

**Authors:** Jan Georg Friesinger, Bente Birkeland, Anne Brita Thorød

**Affiliations:** Department of Psychosocial Health, University of Agder, Kristiansand, Norway

**Keywords:** recovery, animals, atmosphere, human-animal relationship, mental health, ethnography

## Abstract

Being in a relationship with an animal can promote the well-being of people. For many individuals, this usually takes place at home. This study reports about homes for people with mental health problems (with or without co-occurring substance use), who live in supported housing operated by public landlords, entailing tenancies that are usually stricter regarding their pet policies than ordinary homes. We thus addressed the following research questions through ethnographic fieldwork at seven distinct places: which types of human–animal relationships occur in supported housing, and how do they affect the tenants? We analyzed the collected data informed by the Grounded Theory approach and found three types of human–animal relationships within supported housing affecting the tenants differently, namely, “no animals,” “visiting animals,” and “shared/sole ownership of animals.” Animals in the buildings can stage atmospheres that promote solidarity and connectedness among people. In contrast, situations in which animals are forbidden can create emotional tensions between tenants and staff or landlords. When discussing fostering animal atmospheres and limits to keeping pets, we concluded that animals can contribute to the mental health recovery of tenants by creating acknowledgment and rootedness. Therefore, public housing services need to guarantee equal rights to the tenants as they do with every citizen, including the right to keep a pet.

## Introduction

This study reports on a study about human–animal relationships that focus on housing situations for people with mental health problems (with or without co-occurring substance use), who get assistance from community mental health services. This housing approach, termed as “supported housing or accommodations,” can differ from ordinary homes by the built environment (Friesinger et al., 2019a), whereby assisting staff can either be or not be located on-site^1^. The supported housing settings range from care-home-like accommodations to cozy or family-like atmospheres (Friesinger et al., 2020). The research interest for this study lies in the rights and possibilities for the tenants to establish relationships with animals in their homes and how these relationships might matter for their well-being.

In general, the opportunity to establish a relationship with an animal could be an important step for people with mental health problems on their recovery journey (Topor, 2001, p. 285–287). The process of becoming well from mental health problems should thus be considered in light of social and material situatedness (Topor et al., 2011; Duff, 2016), which goes beyond the definition of Anthony (1993) that focuses solely on an individual process. Being in a human–animal relationship thus pertains to a broader situation that includes social, material, and affective aspects relative to how this relationship might be organized within particular places. As such, dwelling places as homes stand out significantly from everyday places where people can share with pets if they can have and afford them. Another issue concerns animal welfare where animals need to be respected and treated well (LMD, 2009; Broom and Fraser, 2015; IAHAIO, 2015; Nussbaum, 2018).

As already mentioned, supported housing is usually organized as tenancy, which means that the right to have a pet is regulated by tenancy policies and agreements. As such, the landlords have the option to deny pets within their rental units. According to the Norwegian legislation, however, tenants are allowed to keep pets without permission if the pets are of no nuisance to the other residents and based on good reasons, such as social and welfare (NOU, 1993, p. 143; KMD, 2009, p. 5–2). In Norway, individuals with mental health problems (with or without co-occurring substance use) are often categorized as people with special housing needs by landlords, which allows them to reduce their tenancy rights by law, unlike ordinary tenancies (KMD, 2009, p. 11). This means that the tenancy agreements in supported housing can be more easily terminated and that tenants have less protection in losing their apartments. As such, a violation of the house rules could allow the landlord to evict a tenant. Andersen et al. (2016) argued that the house rules of supported housing are also commonly restrictive regarding pets, which contradicts both the tenancy legislation (KMD, 2009, p. 5–2) and the concept of citizenship (Rowe, 2015; Rowe and Davidson, 2016), which outlines the rights gap between tenants in supported housing and public landlords.

In a North American case study about supported housing (Hunt and Stein, 2007), the authors advocate for a pet policy that normalizes the situation of keeping a pet for tenants while meeting the concerns of staff and landlords. Furthermore, Hunt and Stein (2007) highlighted the following important themes for tenants who keep pets: “connectedness,” “responsibility,” and “emotional stability.” In a recent study, Fossey et al. (2020) found that pets help people with mental health problems to cope with experienced loneliness at home and promote companionship. From a broader view, housing studies about (older) people experiencing homelessness, disabilities, and low income showed discriminations and insecurities for companion animals regarding their tenancies (Power, 2017; Toohey and Krahn, 2018; McCabe et al., 2021).

Notwithstanding, it would be interesting to know more about the different types of human–animal relationships in supported housing and how they affect the people mostly linked to this situation, i.e., the tenants. Therefore, in this study, we wanted to grasp the human–animal relationships in supported housing from an ethnographic view.

### Animals, Well-Being, and Relationships

It is well-documented that interactions and relationships with animals can have significant effects on the well-being of people, both physical and mental (Serpell, 1991; Berget, 2006; Friedmann et al., 2015). Living with a pet may have positive effects on cardiovascular health and may in some circumstances reduce stress, depression, and anxiety (Friedmann et al., 2015; Brooks et al., 2018; Friedman and Krause-Parello, 2018). Knowing that physical and mental health are inter-connected, we concluded that for many people, contact with animals has the potential to have positive effects on both health and quality of life. Wisdom et al. (2009, p. 430) indicated four elements involved in the recovery process of people connected to animals: (1) providing empathy and “therapy;” (2) providing connections that can assist in redeveloping social avenues; (3) serving as “family” in the absence of or in addition to human family members; and (4) supporting self-efficacy and strengthening a sense of empowerment. People with disabilities and psychosocial problems, and elderly people, among others, often experience loneliness and shrinking social networks. In these situations, a pet can help to compensate, as it provides contact, support, and amusement (Hart and Yamamoto, 2015), which is also applicable to people experiencing homelessness (Kerman et al., 2019).

In addition, living with an animal gives structure to life. Feeding, cleaning cages, and dog walking are all valuable daily routines. For socially secluded individuals, an animal can be a mean to social interaction that may result in higher social capital (Wood et al., 2005). Talking about an animal is less threatening than exposing oneself in an interaction (Power, 2013).

### From Recovery Atmospheres to Animal Geography

As already outlined, Topor et al. (2011) posited that recovery is a process of becoming well from mental health problems that is more than an individual journey, which entails seriously taking the sociomaterial aspect of recovery processes into account (Topor et al., 2020; Larsen et al., 2021). As such, the wide concept of “atmosphere” (Anderson, 2009; Böhme, 2017) might be useful. On the one hand, the concept underlines both the human and non-human parts that stage a particular atmosphere; on the other hand, it describes how space feels like a phenomenon. Duff (2016) used affective atmospheres to explain situations in which people might recover from their mental health problems, which go beyond explanations that are based on psychosocial functioning. He identified three themes of recovery atmospheres, namely, sociality, safety, and belonging, as well as hope and belief. For example, such recovery atmospheres could be linked to architectural settings at supported housing (Friesinger, 2020; Friesinger et al., 2020) or, more generally, promoting for patients at hospitals (Martin et al., 2019; Brown et al., 2020).

We might ask how do animals then appear in such affective atmospheres. The answer is linked to the broader animal geography of places such as the ones described by Philo and Wilbert (2000), which range from wild to rural or urban places. In those places, particular “atmospheres of animals” are staged (Lorimer et al., 2017), which impact the people who are in human–animal relationships. It is important to notice that people are not passively affected but contribute with their practice to the effects (Bille and Simonsen, 2021), which is a matter of all elements that stage the atmospheres, including animals.

## Methods

To examine the human–animal relationships in supported housing and their affection, the first author conducted a multisited ethnography (Marcus, 1995), which was informed by Grounded Theory (GT) (Charmaz, 2014). This study was part of a larger project^2^ that aimed to grasp materialities and the living situation within supported housing (Friesinger et al., 2019b, 2020; Friesinger, 2020). Animals were thereby an important subject already early in fieldwork that was further elaborated with theoretical sampling (Charmaz, 2014; Charmaz and Thornberg, 2020).

The ethnographic research ranged over a 6-month period (between 2016 and 2017) with a stay period of 1 to 2 weeks in a location at different times. Access to the seven different places in the south of Norway was granted by the local community mental health services. All participants signed informed consent forms explaining the study, its aim, and the fact that anonymity was guaranteed. Fieldwork was basically conducted in public/shared spaces of the places. However, some participating tenants did also invite the first author into their individual homes. Out of all the recruited participants of the larger research project, 30 (12 tenants, 24 staff, and four managers) participants, i.e., those who opposed to pets and those who welcomed them, were included in this study on human–animal relationships. The empirical data consisted of participant observations and ethnographic interviews, which were documented by field notes, interview transcripts, and photographs of the places.

Regarding the supported housing settings (Friesinger et al., 2019b), the seven places were operated by public landlords for people with mental health problems (with or without co-occurring substance use). Their locations were mainly semirural, with the 24/7 presence of on-site staff, and the building design highly resembled congregate settings (McPherson et al., 2018), with either small houses or apartments.

The analysis of the empirical data was informed by the GT version of Charmaz (2014) entailing going back and forth between the steps of data collection and analyses. We started with initial coding, followed by focused coding, and finally built our concept with theoretical coding, resulting in three different observed human–animal relationships that affected the people within supported housing: “no animals,” “visiting animals,” and “shared/sole ownership of animals.” These steps were carried out with the help of the software ATLAS.ti (Friese, 2019), whereby we used strategies such as memo writing and constant comparison. The shorter stays in the field with multisited ethnography made theoretical sampling sometimes challenging but was compensated through the various views which it involved. Although our findings primarily account for the sample of the study, they can be applied to similar housing or care settings beyond Norway. We pursued GT quality criteria for our study, such as “credibility” and “usefulness” (Charmaz and Thornberg, 2020), and reflected on any ethical issue that could arise.

## Findings

Our ethnographic research addresses the question of which types of human–animal relationships occur in supported housing and how they affect the people linked to this situation. Our findings point out the following three human–animal relationships regarding tenants of supported housing: “no animals,” “visiting animals,” and “shared/sole ownership of animals.” The human–animal relationships are inter-sections between different elements: people, animals, places, and regulations (including the broader situation). In sum, people can be tenants, staff, and managers who possess different biographies and resources; animals can be wild birds or pets such as dogs or cats; places can be buildings with different architectural designs; and regulations can be pet policies and house rules.

Each of the three human–animal relationships affects the tenants in different ways. For example, the presence of animals can stage affective atmospheres in the buildings, whereas situations with the absence of animals can affect the tenants regarding emotional debates, memories, and desires. In the following, we presented each relation and its affection more thoroughly.

### No Animals Welcome

In our research settings, animals were often not present in supported housing due to no-pet policies and some people not wanting them. No-pet policies were linked to institutional healthcare services in which a manager tried to explain by drawing a line between their supported housing units and care homes within the municipality. During fieldwork, some staff denied any institutional linkage and underlined that the housing units were not meant to be psychiatric wards, which meant that “everything [was] allowed in the apartments.” However, according to the staff, an exception was “to keep a pet.”

This pet restriction was literally confirmed by some of the local house rules, but many tenancy agreements conveyed an unclear pet restriction. This ambiguous pet ban was already pointed out by Andersen et al. (2016). Nonetheless, some of the tenants reported that they could not risk getting evicted if their pets were not clearly approved. This avoidance to keep a pet has to be observed in light of the introduced context, whereby tenants of supported housing have fewer rights and less protection concerning their tenancies than ordinary ones (KMD, 2009, p. 11).

No-pet policies in supported housing are also explained by possible allergic reactions. A manager stated that “the no-pet rule is generally justified because we need to include recent societal developments, whereas people are allergic for several reasons, and we need to consider this also within supported housing.” For example, an allergic staff was used as a reason in one case in which a tenant was denied a dog who criticized the issue as insubstantial because “people could else pollute the indoor environment such as by smoking.” Another manager explained that pets are inappropriate in supported housing because people are physically close to each other without an opportunity for social distancing. Furthermore, the housing unit is a workplace. In this case, the housing setting was designed as congregated apartments, which played an important role: on the one hand, this type of built environment leads to on-site located staff who need a room for themselves to retrieve according to the working environment act; on the other hand, this building design leads to a spatial structure where tenants share common rooms such as dining and living rooms beside their own apartments. In contrast, independent apartments or houses represent more extensive autonomy for the tenants (Friesinger et al., 2019a), whereby allergy-related reasons might play a minor role than in such congregate types.

Nevertheless, some tenants disliked pets such as dogs and did not want co-tenants to get one. For example, some former homeless tenants articulated that they “d[id] not want to have pets around because they [were] too much to deal with.” Similarly, some of the staff informed that pets were restricted because the tenants were not able to take care of pets by themselves and stated: “Otherwise, we need to do it.” Even though a tenant had a pet earlier, such as a guinea pig, the staff needed to apply again by the municipal landlord for them to keep it. The staff expressed for cases similar to this that they “need[ed] to double-check if the tenant [was] capable of keeping animals.”

Overall, several managers and staff defended the no-pet policies and were supported by some tenants who had had negative experiences with pets or cotenants who did not take proper care of their pets. Allergy as a reason to deny pets was thereby linked to the built environment of the supported housing setting.

**Figure 1:**
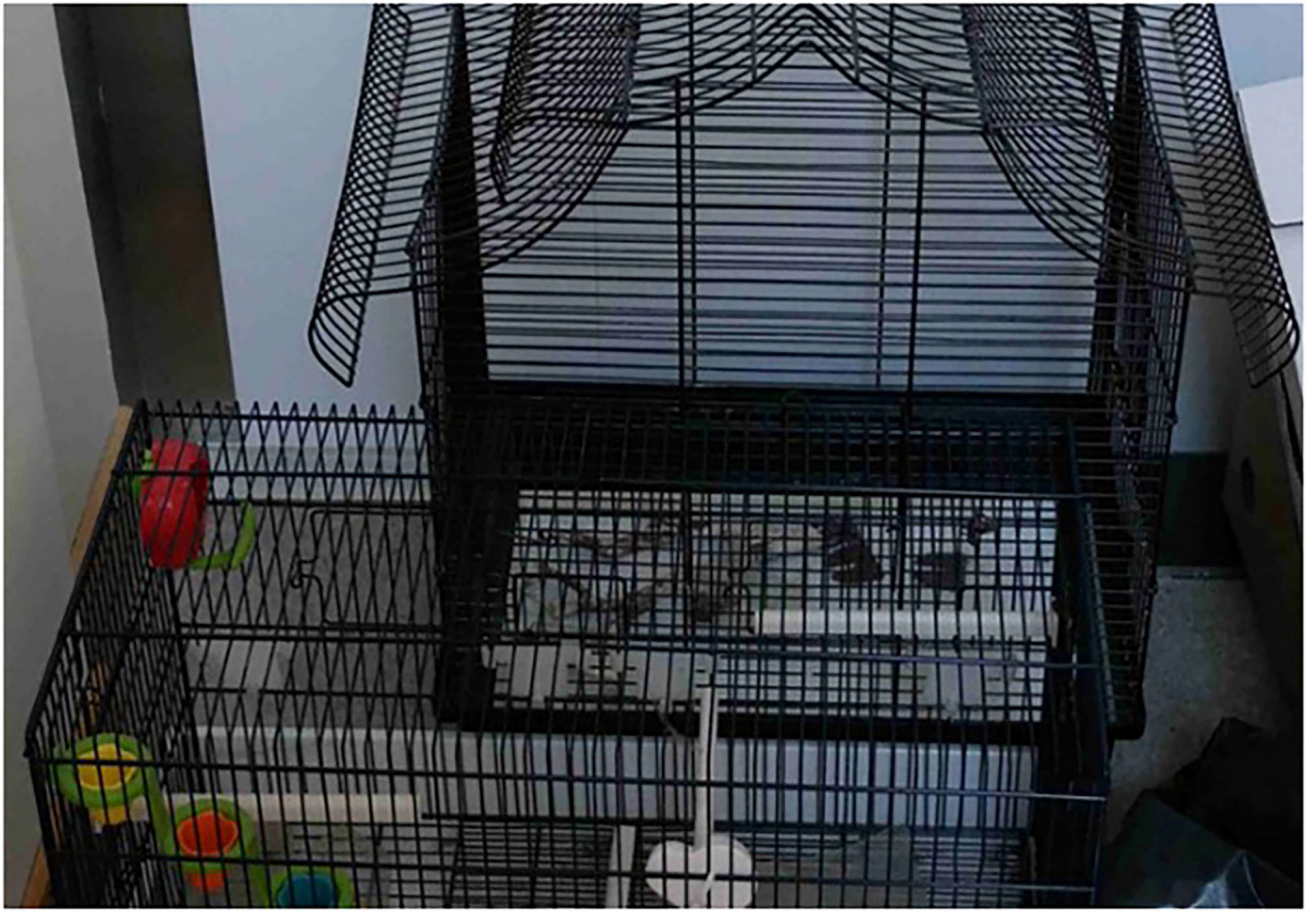
The empty birdcage.

The absence of pets in supported housing due to restrictions was not only an important theme in discussing tenancies but also particularly affected tenants who desired a pet. For example, a tenant who had many nurturing experiences of former human–animal relationships expressed a deep longing for a pet. Furthermore, the tenant highlighted the importance of being able to keep a pet because when you are in a mental health crisis, “a pet connects you to reality.” Other tenants who wanted a pet also expressed the need for a private apartment and the desire to move away from the restrictions. The pet restrictions were also evident in the materials around the places, such as an empty birdcage that a tenant openly positioned before the entrance door (Figure 1) or a large cat pictogram as wall decoration in an apartment which expressed the strong connection of tenant to cats. In both cases, the tenants criticized the pet ban and fought for their rights to have a pet.

In sum, tenants who were denied keeping a pet expressed a general dissatisfaction with their housing situation that was associated with the lack of equal tenancies rights and the feeling of not being respected. Some tenants stood up for their rights to keep a pet and were occasionally supported by staff, while other tenants surrendered.

### Animals That Visit the Place

**Figure 2:**
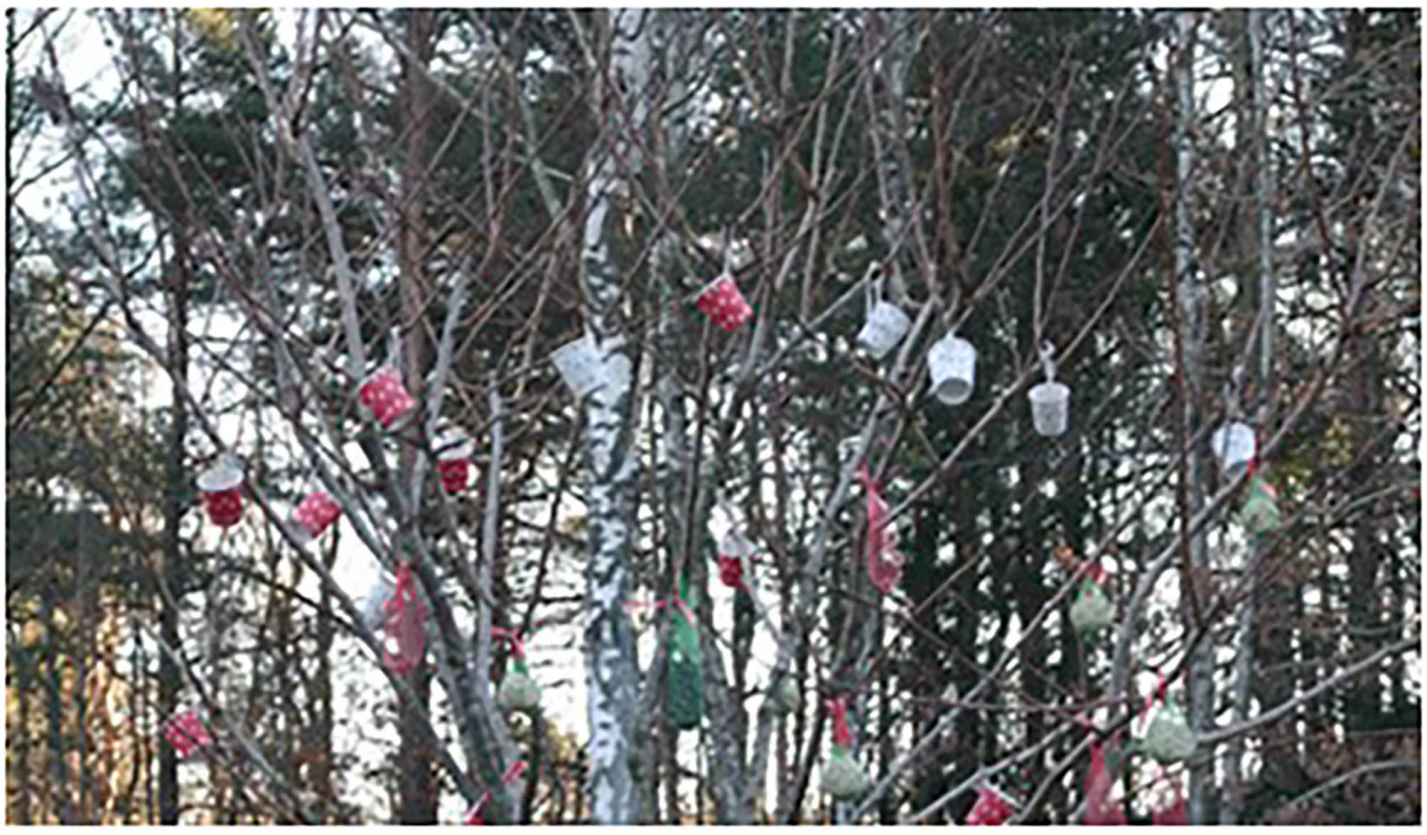
Bird feeders in the garden.

A special type of human–animal relationships occurred when animals visited the place of residence. On the one hand, wild animals such as birds could be found in the outside area of the places; on the other hand, pets owned by others than the tenants came to visit. At several places, there were bird feeders mounted around the housing sites. For example, a staff stated that they made bird feeders together with the tenants and decorated a tree in the garden to create a lively atmosphere (Figure 2). The motives changed according to the annual seasons, and even vegetables were grown at the bottom of the tree. During fieldwork, tenants were observed watching the birds while they smoked or waited outside. Some tenants even organized their own bird feeders to support birds during the wintertime.

Another alternative was that staff who owned pets, such as dogs, brought them to the places so that interested tenants could get in touch with them. Tenants in housing situations with pet restrictions appreciated such visiting pets. A manager positively valued staff who took their own pets with them “because the tenants can thus meet animals even though they do not own one and can get in touch with them.” Despite the benefits of meeting an animal at home, some tenants stressed that this still meant not having equal tenancies rights.

### Shared or Sole Ownership of Pets

The third type of human–animal relationship that appeared in the visited places was animals that were owned either solely by a tenant or by several tenants and staff together. For example, at one residential place, the tenants and staff formerly kept chicken but did not continue after the chicken died due to the additional work to handle them and tenants losing interest. Furthermore, the municipal mental health service reorganization entailed that staff was occupied with other tasks than green care; hence, the chicken cage was abandoned.

Nevertheless, pets could be found where they were shared by the tenants and staff within some of the visited supported accommodations, which entailed a collective responsibility to take care of them. For example, one tenant held the main ownership for a cat, but everybody at the place looked after it, especially when the official owner was admitted to the hospital.

Furthermore, the presence of pets could stage affective atmospheres at the residential places that were appreciated by the tenants. For example, the first author observed a cat walking through the common area of a housing setting with apartments. On its way through the halls, the purring cat was stroked by several tenants who waited for dinner and got a piece of sausage from the staff at the kitchen door. A staff stated within this fellowship: “It is the best therapy to have a cat around,” which was confirmed by the surrounding people. The staff moreover underlined that having a cat meant to be able to take responsibility, whereas a tenant considered the fact that pets were allowed as a key quality of this accommodation. In another congregate setting, cats and dogs were not allowed, whereas fish was welcomed. In a situation where a tenant was agitated, the staff succeeded in calming them down by reminding them to look after their fish.

**Figure 3:**
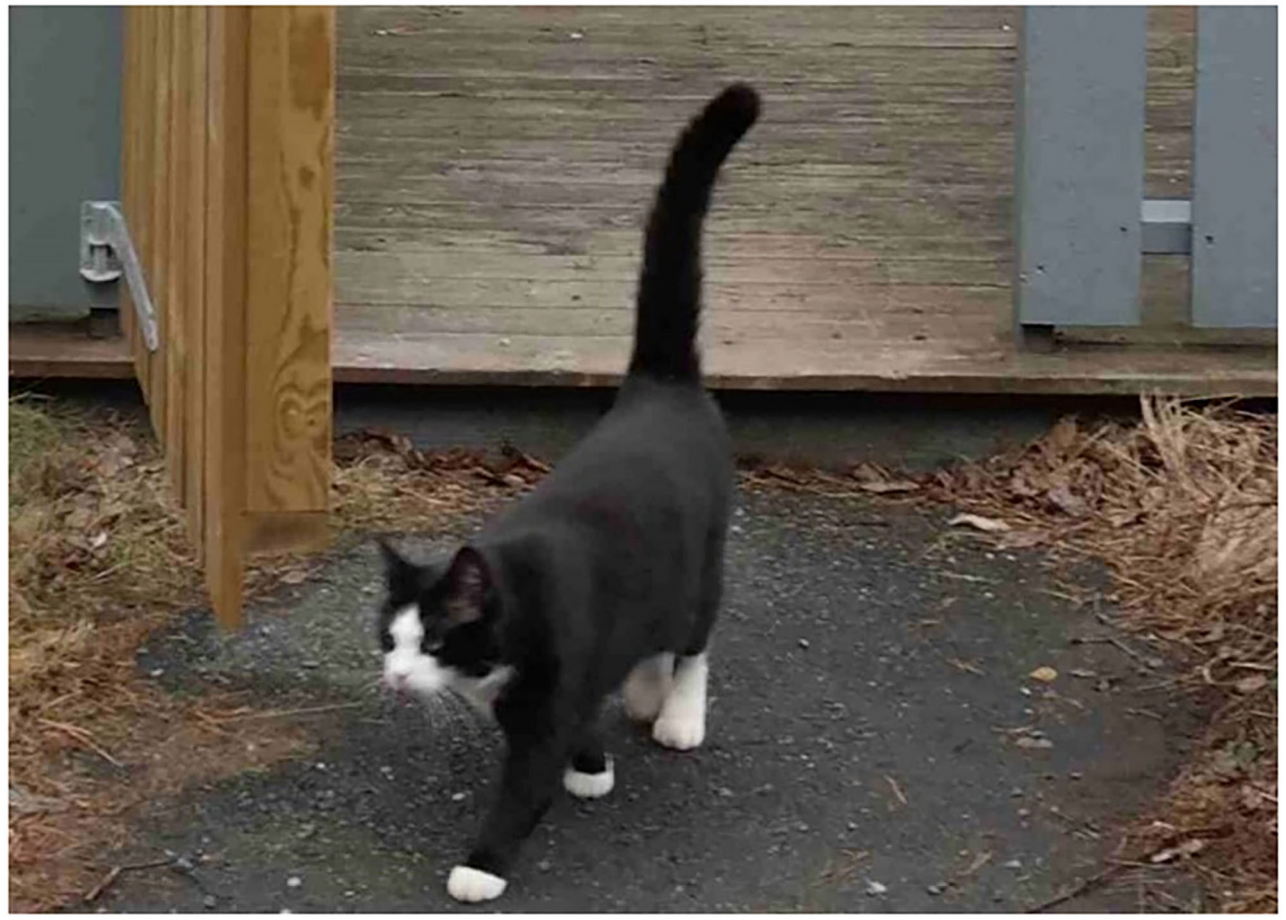
The wandering cat “M”.

Turning to the small colocated houses, some tenants expressed that they own cats and even dogs without any problems. Nevertheless, the pets needed to be approved by the public landlord first. A tenant emphasized the solidarity among tenants in helping each other look after a cat when they were not present. The cat “M” could thereby wander freely around at the place, which not only created an inter-connectedness (fellowship) between people (Figure 3) but also promoted a place attachment in the way the tenant explained: “My beloved cat knows the place best.” The cat lived at the place for more than a decade and was described as extremely cuddly and therapeutic in terms of understanding the condition of the owner, whereby both took care of each other.

## Discussion

The ethnographic research found three types of relationships, namely, “no animals,” “visiting animals,” and “shared/sole ownership of animals,” at supported housing for people with mental health problems (with or without co-occurring substance use). The influences of these three types of human–animal relationships range from positive to negative impacts on people within those places. In the following, we first discussed which positive influences human–animal relationships may have on tenants of supported housing under the lens of recovery atmospheres. Second, we discussed the possible limits to keeping pets in supported housing.

### Fostering Animal Atmospheres

Our findings reveal that the presence of both visiting and owned animals at supported housing can stage, together with the people and the place, a unique affective atmosphere. We might wonder whether these animal atmospheres help foster the mental health recovery of tenants. To this aim, we compared our findings with those relative to the recovery atmospheres introduced by Duff (2016), which involved different issues (i.e., sociality, safety and belonging, as well as hope and belief). To begin with sociality, we observed that tenants and staff described an inter-connectedness that both shared and individual ownership of a cat could establish within the housing. This inter-connectedness revealed a social mutuality that conveyed collective responsibility for the cat beyond the duty of an individual to look after a pet. These acts of solidarity such as caring for the cat “M” by the pet owner and other tenants can be described as “more-than-human solidarity” outlined by Rock and Degeling (2015), in which caring is a broader concern than the commitments of people including other species and places. Nonetheless, the expressions of tenants indicated that a pet such as cat “M” was a social companion that helped coping with loneliness. The so-outlined fostering version of sociality within such animal atmospheres for people with mental health problems can also be found in the introduced literature; for example, pets can create responsibility, social capital, and connectedness (Hunt and Stein, 2007; Brooks et al., 2018; Kerman et al., 2019; Fossey et al., 2020).

Furthermore, the issues of safety and belonging can be identified in the example of the cat that helped create an emotional rootedness to the place by thoroughly knowing the neighborhood over the years. Some tenants stated to feel well or safe with a pet that grounded their life and helped them cope with unpleasant conditions. Similarly, stability for tenants was already an important finding in the study of Hunt and Stein (2007) about pets in supported housing. In other words, pets in supported housing can, together with materialities, stage atmospheres where tenants feel safe at home (Friesinger et al., 2020). Animals are part of a broader geography (Philo and Wilbert, 2000) and, furthermore, as visiting animals, they can contribute to tenants feeling well in their homes, as reported in our findings.

Finally, the issues of hope and belief could be observed both in situations with existing and absent human–animal relationships in our findings. Tenants who longed to have a pet and reported previous experiences of human–animal relationships hoped to get better, to some extent, from their mental health problems with the help of a pet. Additionally, they expressed a belief in life as meaningful, although local pet policies denied them to keep a pet. Visiting animals could thereby underline the belief that changes in life are possible for the tenants in terms of a journey of recovering and managing a life with contradictions (Topor, 2001). However, visiting animals still underscore the rights gap that tenants face in supported housing as a marginalized group (Andersen et al., 2016). Therefore, endorsing pet ownership might contribute to a recovering citizenship (Rowe, 2015; Rowe and Davidson, 2016) in terms of the acknowledgment of the equal rights of tenants in supported housing. In such cases, the tenants in our study expressed a feeling of confidence that was linked to their bond with the pet and to a social identity as pet owners who managed to take care of pets and themselves.

Together, our findings showed that animals, particularly pets, are entangled in recovery atmospheres for tenants in supported housing. It is important to highlight with the study by Bille and Simonsen (2021) that atmospheres, in general, are not static phenomena but involving practices. This means for supported housing that the fostering of animal atmospheres needs to be actively orchestrated, especially in light of the existing rights gap for tenants in supported housing. Another question concerns why animal atmospheres are so unique. An answer might be found in the biophilia hypothesis (Wilson, 1984), i.e., the hypothesis that humans are naturally bonded to nature and, as such, to animals, which explains the impact of animals on the well-being of people (Friedmann et al., 2015). Other explanations draw on social support in interactions or attachment theories (Fine and Weaver, 2018). However, especially cats, dogs, and horses are outstanding animals because they were domesticated through human history.

Animal welfare and ethics might be another reason why animal atmospheres are unique: they remind humans to principles that we treat each other with respect. The foundation of animal welfare can range from an anthropocentric approach to a utilitarian or capabilities approach (Nussbaum, 2018). In her capabilities approach, Nussbaum (2018) advocated for the fundamental rights of animals. In our opinion, tenants of supported housing are more than capable of keeping pets but lack the rights to do so like everybody else. Therefore, capabilities as suggested should be applied to all humans and animals sharing the same world, whereby supported housing should not be an exception.

### Limits to Keeping Pets in Supported Housing

In the context of the recovering bond between humans and animals such as outlined with fostering animal atmospheres, we might ask why more tenants of supported housing do not have pets. Hence, in the following, the potential barriers at the inter-section of societal, organizational, and individual reasons are discussed.

National and local tenancy policies and agreements regulate the rights to have a pet as tenants of supported housing (Hunt and Stein, 2007; KMD, 2009; Andersen et al., 2016). A rights gap is evident between the public landlords and tenants. Our findings confirmed that these landlords are so powerful by law practices (KMD, 2009, p. 11) that the tenants have hardly any chance to argue for a good reason if landlords, together with staff and managers, disagree with them. This rights gap contradicts the fundamental ideas of the equal rights of tenants that are proposed by the models of supported housing and recovering citizenship (Rowe and Davidson, 2016; Sylvestre et al., 2017). Moreover, the assessment by staff and management of whether a tenant is capable of keeping a pet properly or not might be further linked to the practices of stigmatization and marginalization in terms of the tenant being merely seeded as a psychiatric patient (Sayce, 1998, 2016). Tenants from our study reported that they did not feel equally treated, which was not only a matter of paternalistic rules but also the discriminations such as similarly described in other studies about companion animals (Power, 2017; Toohey and Krahn, 2018; McCabe et al., 2021). To compare, people in ordinary tenancies or as homeowners do not need to demonstrate their abilities in advance to keep a pet. However, our findings showed that tenants in supported housing could keep pets properly by themselves or together without any problems while conforming to animal welfare principles (IAHAIO, 2015).

Nevertheless, Berget et al. (2018) pointed out that animal welfare is a hindrance for people who are afraid of the responsibility involved if the pet gets sick. As such, the guidelines as suggested by Hunt and Stein (2007) might be a solution. Specifically, staff could help tenants in animal welfare issues, and we showed that the staff and the tenants kept a cat together in some places. However, we could interpret our study cases in a way that public landlords, managers, and staff might misuse animal welfare or potential allergic reactions as an excuse not to favor pets in supporting housing even though the applying tenants have good reasons. Community mental health services involving public landlords should aim to promote the well-being of people (Prilleltensky, 2005) and therefore need to facilitate that tenants can keep pets in supported housing. However, community services could help to address several challenges of providing pet-friendly housing that is affordable for tenants by addressing the needs of both people and their pets (Toohey et al., 2017).

The proposal of a tenant to keep a pet should be considered individually both in light of animal welfare principles and good reasons such as benefits of the human–animal relationship on the physical and psychosocial health (Wisdom et al., 2009; Friedmann et al., 2015; Brooks et al., 2018; Friedman and Krause-Parello, 2018). As such, Section 11 in the Norwegian Tenancy Act (KMD, 2009) or similar reductions in tenancy rights should be considered terminated because they produce inequalities by dividing tenants into first- and second-level classes concerning their rights. Argumentations that staff might be allergic to pets should have minor weight in considering a proposal of tenants. Hence, the interest of employees in and their attitude to animals in homes should be emphasized in staff employment. The tenants should, moreover, have real chances to appeal the decisions of the landlords. These recommendations would strengthen the rights of tenants toward a recovering citizenship (Rowe, 2015; Rowe and Davidson, 2016) in which people are understood and treated as equal. For example, Zimolag and Krupa (2009) showed that people with continuous mental health problems, who own a pet, had higher social community integration.

Arguing that visiting animals could be a compromise still entails an asymmetric relation in which professionals are in charge. However, tenants with low resources or who are not often at home might benefit from the human–animal relationship based on the shared ownership of a pet. Finally, materialities could be better designed to allow and endorse pets in supported housing, such as cat doors or house designs that reduce nuisance by installing soundproof walls. The findings outlined that building designs that involved a high degree of independence and autonomy were better in facilitating pets. The latter must be observed in light of housing location and neighborhood qualities, including people, animals, and materials (Philo and Wilbert, 2000; Lorimer et al., 2017; Friesinger et al., 2019a).

An individual issue is that not all people are fond of pets and do want one, which needs to be considered by landlords as well but not solely (Toohey et al., 2017). Another issue concerns the suitability of animal type linked to the housing situation of a person. It seems that in our Norwegian settings cats were more likely to be found; a possible reason could be less noise and the fact that they are easier to handle, as shown by our examples. In sum, limits to keeping pets in supported housing are complex and linked, on the one hand, to local social practices at the residential places and, on the other hand, to cultural and political frames for people with mental health problems still involving exclusion tendencies (Sayce, 2016).

## Conclusion

Our study pointed out that in supported housing for people with mental health problems (with or without co-occurring substance use), relationships with no animal, visiting animals, and animals that were shared or solely owned were present. While tenants expressed a general dissatisfaction in places where animals were not welcomed, places with pets could be associated with fostering atmospheres for mental health recovery. Such fostering animal atmospheres based on the human–animal relationships were the results of an inter-section of people, animals, places, and rules whereby pets could create acknowledgment and rootedness. However, there still are rights gaps between tenants and landlords that go beyond pet allowance, especially when it comes to good reasons for keeping animals. We, therefore, criticized the paternalistic ideas and stressed the strengthening of the rights of tenants of public housing, which include keeping pets in compliance with animal welfare toward a recovering citizenship (Rowe and Davidson, 2016).

## Data Availability Statement

The original contributions presented in the study are included in the article/supplementary material, further inquiries can be directed to the corresponding authors.

## Ethics Statement

The studies involving human participants were reviewed and approved by NSD (Norwegian Centre for Research Data). The participants provided their written informed consent to participate in this study.

## Author Contributions

JF contributed to conception and design of the study and collected the data. JF and AT performed the data analysis. JF wrote the first draft of the manuscript and both AT and BB wrote sections of the manuscript. All authors contributed to manuscript revision, read, and approved the submitted version.

## Conflict of Interest

The authors declare that the research was conducted in the absence of any commercial or financial relationships that could be construed as a potential conflict of interest.

## Publisher's Note

All claims expressed in this article are solely those of the authors and do not necessarily represent those of their affiliated organizations, or those of the publisher, the editors and the reviewers. Any product that may be evaluated in this article, or claim that may be made by its manufacturer, is not guaranteed or endorsed by the publisher.

## Footnotes

^1^For more details, McPherson et al. (2018) offered a supported housing classification.

^2^The Norwegian Centre for Research Data, approval number 50067.
